# Medical student debt and major life choices other than specialty

**DOI:** 10.3402/meo.v19.25603

**Published:** 2014-11-11

**Authors:** James Rohlfing, Ryan Navarro, Omar Z. Maniya, Byron D. Hughes, Derek K. Rogalsky

**Affiliations:** 1 Oregon Health and Science University, School of Medicine, Portland, OR, USA; 2 Georgetown University School of Medicine, Washington, DC, USA

**Keywords:** debt, specialty choice, primary care, medical student, salary, burnout

## Abstract

**Background:**

Median indebtedness at graduation is now more than $170,000 for graduates of US Medical Schools. Debate still exists as to whether higher debt levels influence students to choose high paying non-primary care specialties. Notably, no previous research on the topic has taken into account cost of attendance when constructing a debt model, nor has any research examined the non-career major life decisions that medical students face.

**Methods:**

Medical students were surveyed using an anonymous electronic instrument developed for this study. The survey was delivered through a link included in a study email and students were recruited from school wide listservs and through snowball sampling (students were encouraged to share a link to the survey with other medical students). No incentives were offered for survey completion.

**Results:**

Responses were recorded from 102 US Allopathic medical schools (*n*=3,032), with 22 institutions (11 public, 11 private) meeting inclusion criteria of 10% student body response proportion (*n*=1,846). Students with higher debt relative to their peers at their home institution reported higher frequencies of feeling callous towards others, were more likely to choose a specialty with a higher average annual income, were less likely to plan to practice in underserved locations, and were less likely to choose primary care specialties. Students with higher aggregate amounts of medical student loan debt were more likely to report high levels of stress from their educational debt, to delay getting married and to report disagreement that they would choose to become a physician again, if given the opportunity to revisit that choice. Increases in both aggregate and relative debt were associated with delaying having children, delaying buying a house, concerns about managing and paying back educational debt, and worrying that educational debt will influence one's specialty choice.

**Conclusions:**

Medical student debt and particularly debt relative to peers at the same institution appears to influence the way that students approach major life choices like when to start a family, when to buy a home, and what specialty to choose. Future research should take into account cost of attendance when looking for the impact of medical student debt on major life choices.

In 1992, the median indebtedness at graduation for medical students was $50,000. Twenty years later in 2012, this figure was $170,000 (1). Even when inflation is accounted for, this represents a dramatic increase in the cost of medical education to the student. The rise in cost and debt has coincided with an ever-growing shortage of primary care (PC) physicians and a decline in the monetary return on investment for a career in PC (2). This has prompted leaders in medical education to propose new models of education that reduce time in training and rely on competency-based approaches rather than a time in training model (3, 4). However, the high and rising median debt does not tell the whole story. From 2002 to 2012, the number of students graduating with no debt decreased from 17 to 15%, while the number of students graduating with over $200,000 in student loan debt increased from 5 to 29% (5). At the same time, the proportion of students matriculating from families with incomes in the bottom quintile has decreased and underrepresented minorities rank cost as the number one deterrent to pursuing a medical education (6, 7). These facts suggest that the high cost of attendance may be negatively influencing diversity in medical schools and that the distribution of medical education debt is becoming ever more skewed.

While the long-term consequences of rising debt and decreasing diversity are yet to be fully realized, one large study, among internal medicine residents, demonstrated that higher levels of educational debt are positively associated with burnout and negatively associated with quality of life and standardized test scores (8). This suggests that debt may already be weighing on the ability of some residents to learn and perform at a high level.

Despite these disturbing trends of increasing inequality, decreasing diversity, and the suggestion that debt may already be weighing down young physicians, debate still exists in the literature as to how influential medical student debt is on specialty choice. While some studies have found no correlation between absolute debt level and specialty choice (9–11), others have found modest correlations (12). One particularly instructive study found that increasing debt level may help to predict which first year medical students will change their plans from PC to a high paying non-PC specialty (HPNP) by the end of 4 years of medical school (13). These previous thoughtful analyses and conflicting conclusions suggest that specialty choice is a complex, personal, and multifactorial decision (14).

Interestingly, all previous studies, both single and multi-institutional, have used an absolute debt model, where the number of dollars owed was used directly as a determinant in analyses, regardless of differences in cost of attendance at different institutions. This model leaves out important information, as medical students may be far more aware of and therefore influenced by their debt relative to the cost of attendance of their respective institution and not a distant national average. Indeed, there is ample evidence from psychological experiments and medical studies that support the idea that value may be more of a relative measure that changes depending on context (15, 16). It therefore behooves analyses on the impacts of student loan debt including multiple institutions to consider the effects of both relative and absolute debt for analyses.

Moreover, there have been no previous studies looking at major life decisions among medical students other than specialty choice, such as when to buy a home, get married, or have children. While major life decisions other than specialty choice may be considered less relevant to national health policy, delaying or foregoing these other life events may be a significant source of frustration, stress, and burnout among medical students, residents and physicians. Burnout among medical students has been associated with an increased likelihood of engaging in unprofessional conduct and a decreased likelihood of holding altruistic views regarding physicians’ responsibilities to society (17). Similarly, alarming associations with burnout have been found among residents and physicians, including decreased standardized test scores for residents and increased medical errors among practicing physicians (8, 18, 19). Therefore, we set out to study medical student specialty choices and major life decisions in the context of both absolute and relative debt levels at multiple institutions across the nation. Drawing on the aforementioned research and our own experience, we hypothesized that allopathic US medical students with increasing levels of absolute and relative debt would be more likely to report delaying or planning to delay buying a home, getting married, and having children. We also hypothesized that they would be more likely to report frequent feelings of burnout, as well as stress and debt management concerns. We hypothesized that these increased levels of relative and absolute debt would also translate into reporting a decreased desire to practice in rural, underserved, and inner-city areas or become a physician again if given the opportunity. We also hypothesized that relative debt more than absolute debt would predict which students chose HPNP specialties.

## Methods

The Georgetown University Medical Center Institutional Review Board approved this study and waiver of informed consent. The Oregon Health and Science University Institutional Review Board determined this project to be not human subject research. An anonymous questionnaire was developed, using Google Forms^®^, and distributed electronically by email to all currently enrolled medical students at specific medical schools based on convenience. The respondents self-reported current levels of debt, scholarships, and enrollment in service scholarships as well as their demographic information and responses to the study items (Tables 1–3). An assessment of burnout was conducted using two questions that correlate strongly with the Maslach Burnout Index (20). The survey was piloted with a sample of convenience of 20 medical students, equally divided by year in school and gender. Based on qualitative feedback, items were revised. Several specific individuals from a geographically diverse group of public and private medical schools were approached to distribute the survey at their institution and encourage a higher response proportion by sending the survey through email multiple times over the study period (February 2013–May 2013). Snowball sampling was also employed and students were encouraged to distribute a survey hyperlink to their colleagues by email. No monetary or other prize was offered as an incentive.

**Table 1:** Comparison of included respondent demographics to national data on medical students

Demographics	Observed	Expected
Percent male	47.4	53.2
Percent female	52.6	46.8
Percent American Indian or Alaskan Native	0.4	0.8
Percent Asian or Asian American	12.1	22.5
Percent Black or African American	3.5	6.9
Percent Native Hawaiian or other Pacific Islander	0.4	0.3
Percent Hispanic	5.0	8.8
Percent White	79.6	59.2
Percent some other race	3.0	0.0
Percent No answer or Prefer not to answer	4.9	1.9
Percent private attendance	50.4	40.2
Percent public attendance	49.6	59.8
Percent 1st year	27.1	Not available
Percent 2nd year	23.9	Not available
Percent 3rd year	20.7	Not available
Percent 4th year	23.4	Not available
Percent 5th year	3.2	Not available
Percent 6th year	0.7	Not available
Percent 7th year	0.3	Not available
Percent 8th year	0.1	Not available
Percent 9th year	0.2	Not available
Self-reported median debt		
First year	43,300	Not available
Second year	96,000	Not available
Third year	150,000	Not available
Fourth year	200,000	Not available
Fifth year	200,000	Not available
Sixth year	200,000	Not available
Seventh year	160,000	Not available
Eighth year	7,500	Not available
Ninth year	37,500	Not available
2013 graduates	200,000	Not available
Mean 2013 graduating debt	188,838	135,084

**Table 2:** Comparison of included medical school demographics to national medical school demographics

Demographics	Included schools	All schools
Percent Male	52.8	53.2
Percent Female	47.2	46.8
Percent American Indian or Alaskan Native	0.8	0.8
Percent Asian or Asian American	22.1	22.5
Percent Black or African American	5.1	6.9
Percent Native Hawaiian or other Pacific Islander	0.8	0.3
Percent Hispanic	5.9	8.8
Percent White	65.0	59.2
Percent Some other race	0.0	0.0
Percent No answer or Prefer not to answer	1.4	1.9
Percent private attendance	55.5	40.2
Percent public attendance	44.5	59.8

**Table 3:** Comparison of respondent demographics to respective medical school demographics

	Responses			Male		Female		White		Black		Hispanic		Asian		Native American		Pacific Islander		Other		No response	
											
School	Actual	Expected	Percent	Actual percent	Expected percent	Actual percent	Expected percent	Actual percent	Expected percent	Actual percent	Expected percent	Actual percent	Expected percent	Actual percent	Expected percent	Actual percent	Expected percent	Actual percent	Expected percent	Actual percent	Expected percent	Actual percent	Expected percent
Albany Medical College	75	580	12.9	48	54	52	46	76	51	3	5	7	4	23	34	0	1	0	0	4	0	3	4
Baylor College of Medicine	96	827	11.6	46	54	54	46	66	47	8	5	14	14	21	36	2	1	0	0	5	0	5	0
Boston University School of Medicine	107	795	13.5	40	49	60	51	65	44	7	7	9	12	19	32	0	0		0	7	0	7	5
George Washington University School of Medicine and Health Sciences	95	734	12.9	41	46	58	54	74	53	4	10	2	4	16	29	1	0	1	0	5	0	7	2
Georgetown University School of Medicine	210	822	25.5	43	51	57	49	78	67	4	6	4	3	13	21	0	0	0	0	2	0	2	1
Louisiana State University School of Medicine in Shreveport	59	478	12.3	53	55	47	45	86	81	3	4	2	3	5	9	0	0	0	0	3	0	2	2
Medical College of Wisconsin	108	842	12.8	50	56	50	44	86	70	4	5	6	5	9	20	1	2	0	0	2	0	1	1
Northeast Ohio Medical University	62	522	11.9	60	55	40	45	74	58	0	2	0	2	18	35	0	1	0	0	6	0	5	2
Oregon Health & Science University School of Medicine	186	551	33.8	45	48	55	52	86	77	1	1	3	4	8	17	0	1	1	0	3	0	6	1
Rush Medical College of Rush University Medical Center	74	563	13.1	41	50	59	50	80	61	1	5	8	11	14	21	1	1	0	1	3	0	5	0
Southern Illinois University School of Medicine	32	306	10.5	56	52	44	48	81	75	6	11	0	4	6	11	0	0	0	0	0	0	3	0
Stanford University School of Medicine	65	462	14.1	45	51	55	49	57	39	9	5	11	11	22	40	0	1	0	1	6	0	6	5
The Commonwealth Medical College	39	267	14.6	51	57	49	43	82	70	3	5	3	5	13	21	0	1	3	0	3	0	5	0
Tulane University School of Medicine	142	775	18.3	54	58	46	42	84	70	3	2	4	3	9	20	1	1	1	0	4	0	1	2
University of Central Florida College of Medicine	35	278	12.6	43	49	54	51	74	59	6	6	14	14	23	23	0	2	3	0	6	0	3	0
University of Connecticut School of Medicine	40	386	10.4	30	49	70	51	70	61	8	10	3	6	13	19	0	1	0	0	3	0	8	2
University of Louisville School of Medicine	84	665	12.6	46	55	50	45	80	80	4	6	4	2	8	11	0	0	1	0	2	0	10	1
University of Mississippi School of Medicine	57	527	10.8	56	60	44	40	89	80	5	11	2	1	4	7	0	1	0	0	0	0	2	0
University of Missouri–Columbia School of Medicine	77	410	18.8	45	52	55	48	91	81	0	3	5	3	3	12	1	2	0	0	0	0	5	0
University of Utah School of Medicine	72	348	20.7	64	66	36	34	92	79	1	1	6	6	6	14	0	0	0	0	3	0	4	1
University of Washington School of Medicine	104	972	10.7	40	46	60	54	88	75	2	2	4	7	9	17	1	1	0	1	4	0	6	0
Virginia Tech Carilion School of Medicine	27	126	21.4	70	63	30	37	85	75	0	0	0	0	11	21	0	2	0	0	0	0	4	1

The data was exported from Google Forms^®^ into Microsoft Excel^®^ and Stata SE 12.1 for Windows for subsequent analyses. Responses to categorical Likert scale questions were grouped into categories of ‘Agree’, ‘Disagree’, and ‘Neutral’. For simplicity, our calculations only compare respondents who expressed a deliberate opinion of agree or disagree. Data were imputed from text where necessary (e.g., $50K=$50,000) and excluded when deemed erroneous. Errors constituted the vast minority of entries.

In order to evaluate the effects of both relative and absolute debt on the outcomes of interest, we needed to create a quantitative estimate of the relative debt of each respondent. This was calculated as the proportion of estimated cost of attendance saved (PECOAS). To calculate the PECOAS, the estimated cost of attendance was calculated as the average of the 2010–2011 and 2011–2012 resident (in-state) tuition, fees, and health insurance costs for each respondent's respective school and then multiplied by the number of years he/she had been in attendance. The self-reported medical student loan debt was then subtracted from this estimated cost of attendance, and the difference was subsequently divided by the estimated cost of attendance to yield the PECOAS. Positive PECOAS values correspond to owing less than would be expected given the respondent's institution and year in medical school, while negative values correspond to owing more than would be expected.

## Results

The survey collected 3,032 responses from 102 allopathic medical schools. Of these, 22 schools, 11 public, 11 private reached the threshold of 10% or greater response proportion, leading to an inclusion group of *n*=1,846 and an overall response proportion of 15%, individually ranging from 33.8 to 10.4% (Tables 1–3) (21–23). Mean PECOAS and medical student debt levels for those who agreed and disagreed to each survey item can be found in Table 4.

A multivariate logistic regression of question 1 (I have already or may in the future delay buying a house because of my educational debt) revealed that the presence of consumer debt (Adjusted OR 1.44, 95% CI: 1.02, 2.02), each increase in pre-medical debt by $20,000 (Adjusted OR 1.16, 95% CI: 1.04, 1.30), each additional year in medical school (Adjusted OR 0.86, 95% CI: 0.74, 0.999), each decrease in relative debt as measured by an increase in the PECOAS by one (Crude OR 0.36, 95% CI: 0.29, 0.45, Adjusted OR 0.50, 95% CI: 0.35, 0.71), and each increase in medical student loan debt by $50,000 (Crude OR 1.46, 95% CI: 1.32, 1.61, Adjusted OR 1.30, 95% CI: 1.08, 1.56) were all associated in a statistically significant way with agreement with the above statement.

A multivariate logistic regression of question 2 (I have already or may in the future delay getting married because of my educational debt) revealed that each increase in pre-medical debt by $20,000 (Adjusted OR 1.16, 95% CI: 1.08, 1.25), each additional year in medical school (Adjusted OR 0.67, 95% CI: 0.57, 0.79), identifying as married (Adjusted OR 0.04, 95% CI: 0.02, 0.07), and each increase in medical student loan debt by $50,000 (Crude OR 1.12, 95% CI: 1.05, 1.19, Adjusted OR 1.49, 95% CI: 1.28, 1.73) were all associated in a statistically significant way with agreement with the above statement. Notably, each decrease in relative debt as measured by an increase in the PECOAS by one was not associated with agreement with the above statement (Adjusted OR 1.03, 95% CI: 0.78, 1.37).

A multivariate logistic regression of question 3 (I have already or may in the future put off having children because of my educational debt) revealed that identifying as Black or African American (Adjusted OR 0.47, 95% CI: 0.24, 0.94), the presence of consumer debt (Adjusted OR 1.44, 95% CI: 1.08, 1.91), each increase in pre-medical debt by $20,000 (Adjusted OR 1.14, 95% CI: 1.06, 1.24), each additional year in medical school (Adjusted OR 0.82, 95% CI: 0.71, 0.94), being married (Adjusted OR 0.64, 95% CI: 0.47, 0.89), each additional child (Adjusted OR 0.22, 95% CI: 0.14, 0.33), each decrease in relative debt as measured by an increase in the PECOAS by one (Crude OR 0.51, 95% CI: 0.44, 0.61, Adjusted OR 0.60, 95% CI: 0.45, 0.80), and each increase in medical student loan debt by $50,000 (Crude OR 1.23, 95% CI: 1.14, 1.31, Adjusted OR 1.25, 95% CI: 1.08, 1.44) were all associated in a statistically significant way with agreement with the above statement.

A multivariate logistic regression of question 4 (In the future I plan to practice in an underserved area) revealed that identifying as female (Adjusted OR 2.71, 95% CI: 2.08, 3.54), attending a private medical school (Adjusted OR 0.67, 95% CI: 0.50, 0.91), and each decrease in relative debt as measured by an increase in the PECOAS by one (Crude OR 0.97, 95% CI: 0.83, 1.14, Adjusted OR 1.32, 95% CI: 1.003, 1.74) were all associated in a statistically significant way with agreement with the above statement. Notably, each increase in medical student loan debt by $50,000 was nearly associated with agreement with the above statement (Adjusted OR 1.14, 95% CI: 0.997, 1.30).

A multivariate logistic regression of question 5 (I am likely to practice in an inner-city area upon graduation from residency) revealed that identifying as female (Adjusted OR 1.59, 95% CI: 1.22, 2.08), identifying as Black or African American (Adjusted OR 4.26, 95% CI: 1.60, 11.39), each additional child (Adjusted OR 0.61, 95% CI: 0.42, 0.89), identifying as married (Adjusted OR 0.70, 95% CI: 0.50, 0.99), and attending a private medical school (Adjusted OR 1.63, 95% CI: 1.20, 2.21) were all associated in a statistically significant way with agreement with the above statement. Notably, neither each decrease in relative debt as measured by an increase in the PECOAS by one (Adjusted OR 1.13, 95% CI: 0.86, 1.50) nor each increase in medical student loan debt by $50,000 (Adjusted OR 1.04, 95% CI: 0.90, 1.19) were associated in a statistically significant way with agreement with the above statement.

A multivariate logistic regression of question 6 (Practicing in a rural area interests me) revealed that identifying as Asian or Asian American (Adjusted OR 0.34, 95% CI: 0.21, 0.54), each additional year in medical school (Adjusted OR 0.80, 95% CI: 0.70, 0.92), identifying as married (Adjusted OR 1.68, 95% CI: 1.22, 2.32), and attending a private medical school (Adjusted OR 0.46, 95% CI: 0.35, 0.62) were all associated in a statistically significant way with agreement with the above statement. Notably, neither each decrease in relative debt as measured by an increase in the PECOAS by one (Adjusted OR 1.20, 95% CI: 0.93, 1.55) nor each increase in medical student loan debt by $50,000 (Adjusted OR 1.12, 95% CI: 0.98, 1.28) were associated in a statistically significant way with agreement with the above statement.

A multivariate logistic regression of question 7 (Choose to become a physician again, if you were to revisit that choice) revealed that being married (Adjusted OR 1.89, 95% CI: 1.18, 3.05), attending a private medical school (Adjusted OR 1.78, 95% CI: 1.18, 2.67), and each increase in medical student loan debt by $50,000 (Crude OR 0.86, 95% CI: 0.79, 0.93, Adjusted OR 0.78, 95% CI: 0.66, 0.91) were all associated in a statistically significant way with agreement with the above statement. Notably, each decrease in relative debt as measured by an increase in the PECOAS by one was nearly associated with agreement with the above statement (Adjusted OR 0.71, 95% CI: 0.49, 1.02).

A multivariate logistic regression of question 8 (My educational debt often causes me high levels of stress) revealed that identifying as female (Adjusted OR 1.70, 95% CI: 1.30, 2.23), identifying as Black or African American (Adjusted OR 0.39, 95% CI: 0.20, 0.76), the presence of consumer debt (Adjusted OR 1.72, 95% CI: 1.31, 2.27), each additional year in medical school (Adjusted OR 0.79, 95% CI: 0.69, 0.92), and each increase in medical student loan debt by $50,000 (Crude OR 1.54, 95% CI: 1.43, 1.67, Adjusted OR 1.55, 95% CI: 1.34, 1.81) were all associated in a statistically significant way with agreement with the above statement. Notably, each decrease in relative debt as measured by an increase in the PECOAS by one was nearly associated with agreement with the above statement (Adjusted OR 0.76, 95% CI: 0.57, 1.001).

A multivariate logistic regression of question 9 (I worry about my ability to pay back my educational debt) revealed that identifying as female (Adjusted OR 1.95, 95% CI: 1.46, 2.62), the presence of consumer debt (Adjusted OR 1.92, 95% CI: 1.40, 2.63), each increase in pre-medical debt by $20,000 (Adjusted OR 1.19, 95% CI: 1.07, 1.32), each additional year in medical school (Adjusted OR 0.70, 95% CI: 0.60, 0.82), each decrease in relative debt as measured by an increase in the PECOAS by one (Crude OR 0.27, 95% CI: 0.22, 0.33, Adjusted OR 0.46, 95% CI: 0.33, 0.65), and each increase in medical student loan debt by $50,000 (Crude OR 1.58, 95% CI: 1.45, 1.73, Adjusted OR 1.46, 95% CI: 1.23, 1.74) were all associated in a statistically significant way with agreement with the above statement.

A multivariate logistic regression of question 10 (I am concerned about my ability to manage my educational debt) revealed that identifying as female (Adjusted OR 2.18, 95% CI: 1.65, 2.89), the presence of consumer debt (Adjusted OR 1.66, 95% CI: 1.23, 2.24), each increase in pre-medical debt by $20,000 (Adjusted OR 1.16, 95% CI: 1.06, 1.27), each additional year in medical school (Adjusted OR 0.79, 95% CI: 0.69, 0.92), being married (Adjusted OR 0.69, 95% CI: 0.48, 0.98), each decrease in relative debt as measured by an increase in the PECOAS by one (Crude OR 0.33, 95% CI: 0.27, 0.40, Adjusted OR 0.63, 95% CI: 0.46, 0.85), and each increase in medical student loan debt by $50,000 (Crude OR 1.70, 95% CI: 1.55, 1.86, Adjusted OR 1.68, 95% CI: 1.42, 1.99) were all associated in a statistically significant way with agreement with the above statement.

When asked to report how often they felt burned out by medical school (measuring emotional exhaustion) according to ranked categorical outcomes, only 2.9% of respondents reported never feeling burned out, while 36.6% reported feeling burned out at least four times per month. The average frequency of burnout was approximately 5.6 times per month. These conclusions assume ‘A few’ to be the numerical value of three. A multivariate linear regression revealed that on average being over 30 years of age increased the number of burnout events per month by 1.67 events per month (*p*=0.02, 95% CI: 0.26, 3.07), identifying as female increased the number of burnout events per month by 0.95 events per month (*p*=0.02, 95% CI: 0.16, 1.74), identifying as married decreased the number of burnout events per month by 1.50 events per month (*p*=0.004, 95% CI: −2.52, −0.48), and that attending a private medical school decreased the number of burnout events per month by 1.30 events per month (*p*=0.005, 95% CI: −2.20, −0.39). Notably, neither each decrease in relative debt as measured by an increase in PECOAS by one (*p*=0.54, 95% CI: −1.06, 0.55) nor each increase in medical student loan debt by $50,000 (*p*=0.33, 95% CI: −0.20, 0.60) were associated with changes in burnout frequency.

When asked to report how often they felt that they had become more callous toward people since beginning medical school (measuring depersonalization) in ranked categorical outcomes, 15.1% of respondents reported never feeling more callous, while 26.5% reported feeling more callous at least four times per month. The average frequency of feeling more callous was approximately 4.1 times per month. These conclusions assume ‘A few’ to be the numerical value of three. A multivariate linear regression revealed that on average each additional year in medical school increased the number of callous events per month by 0.61 events per month (*p*=0.001, 95% CI: 0.24, 0.99), identifying as married decreased the number of callous events per month by 1.19 events per month (*p*=0.008, 95% CI: −2.08, −0.31), attending a private medical school decreased the number of callous events per month by 0.80 events per month (*p*=0.048, 95% CI: −1.59, −0.01), and each decrease in relative debt as measured by an increase in the PECOAS by one decreased the number of callous events per month by 0.70 events per month (*p*=0.050, 95% CI: −1.41, −0.001). Notably, each increase in absolute medical student loan debt by $50,000 was not associated with changes in callous frequency (*p*=0.80, 95% CI: −0.30, 0.39).

When asked to report what salary respondents would need after residency to live the lifestyle they desire and pay down their educational debt, 68.3% of respondents selected an annual salary of less than $300,000, while just 3.9% selected an annual salary greater than $500,000. A multivariate linear regression revealed that on average identifying as female decreased the desired salary by $37,000 (*p*<0.001, 95% CI: −$47,000, −$26,000), each increase in pre-medical student loan debt by $20,000 increased the desired salary by $4,500 (*p*<0.001, 95% CI: $2,100, $6,900), each additional year in medical school decreased the desired salary by $10,000 (*p*<0.001, 95% CI: −$16,000, −$4,700), being married decreased the desired salary by $16,000 (*p*=0.018, 95% CI: −$30,000, −$2,900), being divorced decreased the desired salary by $53,000 (*p*=0.041, 95% CI: −$104,000, −$2,100), attending a private medical school increased the desired salary by $18,000 (*p*=0.004, 95% CI: $5,500, $30,000), and each increase in medical student loan debt by $50,000 increased the desired salary by $13,000 (*p*<0.001, 95% CI: $7,800, $18,000). Notably, each decrease in relative debt as measured by an increase in the PECOAS by one was nearly associated with a decrease in desired salary by $10,000 (*p*=0.066, 95% CI: −$21,000, $670).

Respondents were asked to select what medical specialty they would chose if they were forced to choose today. Data on the median physician salary for each specialty chosen were then correlated with the selected specialty (24). Respondents who selected ‘Internal Medicine Subspecialty’ and ‘Pediatrics Subspecialty’ were excluded from this analysis because of the widely varying financial compensation within these groups. This resulted in a reduction to 84.13% of respondents being included (*n*=1,542). Where median salary for all physicians was unavailable, the median associate professor salary was substituted. A multivariate linear regression revealed that on average identifying as female decreased the salary of the desired specialty by $61,000 (*p*<0.001, 95% CI: −$75,000, −$48,000), identifying as Asian or Asian American increased the salary of the desired specialty by $36,000 (*p*=0.001, 95% CI: $16,000, $57,000), attending a private medical school increased the salary of the desired specialty by $32,000 (*p*<0.001, 95% CI: $16,000, $47,000), and each decrease in relative debt as measured by an increase in PECOAS by one decreased the salary of the desired specialty by $21,000 (*p*=0.003, 95% CI: −$34,000, −$6,900). Notably, each increase in medical student loan debt by $50,000 was not associated in a statistically significant way with changes in the salary of the desired specialty (*p*=0.09, 95% CI: −$13,000, $840).

The selected physician specialties, including internal medicine subspecialty and pediatric subspecialty, were then grouped according to PC or not primary care (NPC). PC specialties were defined as pediatrics, geriatrics, psychiatry, family or internal medicine, and obstetrics and gynecology. 30.1% of respondents chose a PC specialty, while 69.9% of respondents did not. The mean PECOAS for students who selected PC and NPC specialties were −0.21 and −0.24 respectively, with a *p*-value of 0.19. The mean medical student loan debt amount for students who selected PC and NPC specialties were $120,000 and $115,000 respectively, with a *p*-value of 0.10. A multivariate logistic regression revealed that identifying as female (Adjusted OR 3.77, 95% CI: 2.94, 4.82), each increase in pre-medical student loan debt by $20,000 (Adjusted OR 0.91, 95% CI: 0.85, 0.97), attending a private medical school (Adjusted OR 0.67, 95% CI: 0.51, 0.87), and each decrease in relative debt as measured by an increase in PECOAS by one (Crude OR 1.06, 95% CI: 0.93, 1.23, Adjusted OR 1.29, 95% CI: 1.01, 1.65) were all statistically significantly associated with choosing a PC specialty. Notably, each increase in medical student loan debt by $50,000 was nearly associated with the choice of a PC specialty (Adjusted OR 1.12, 95% CI: 0.997, 1.261).

## Discussion

The influence of student debt on the career decisions of medical students, young physicians, and ultimately the physician workforce is extremely complex. In this study, we report for the first time on the effects of student debt on major life choices other than specialty and suggest an alternative model for studying and analyzing medical student debt, the relative model.

As we hypothesized, relative debt may better predict professional decisions than absolute debt. While increases in absolute debt were positively associated with increases in explicitly desired future salary, only increases in relative debt were actually associated with choosing a specialty with a higher salary. Furthermore, students with higher levels of relative debt were less likely to choose PC or practice in an underserved area, while there was no association with absolute debt. The association between relative debt and PC specialty choice is corroborated by a 1996 study by Rosenthal et al. that found that medical student loan debt levels greater than $75,000 were negatively associated with PC specialty choice (25). Prima facie this seems to suggest that absolute medical student debt should be negatively associated with PC choice. However, Rosenthal's study reviewed aggregate debt for a number of students at one academic institution (25). Thus, the recorded aggregate medical student loan debt amounts were actually relative debt amounts for that institution similar to the more recent 2012 study by Grayson et al. that also found debt to be associated with choosing a HPNP specialty (13). Therefore, their concluded association between reduced relative debt and PC specialty choice is corroborated by our study.

Further evaluation of our data suggests a surprising phenomenon. Contrary to common perception, respondents in our study with greater than $75,000 in medical student loan debt had an adjusted odds ratio of 1.47 (95% CI: 1.04, 2.06) of choosing a PC specialty. Higher binary cut-offs for aggregate debt amounts did not find a statistically significant association with PC specialty choice, suggesting that while relative debt is negatively associated with PC specialty choice, students with mid-to-high level absolute debt may have an increased odds of choosing PC over students with little debt, though this needs to be further elucidated. This association may come about because some students with a relatively low debt burden are likely students from more wealthy backgrounds who may see higher paying careers as more attractive.

As we hypothesized, there are a number of major non-career life decisions that are affected by debt. Increasing relative debt increased the likelihood of delaying having children, buying a house, and worrying about the ability to pay back and manage educational debt. While the ramifications of these non-career choices may be hard to quantify, it seems likely that the more young physicians are asked to delay or sacrifice for their career, the more likely they are to experience frustration and burnout. It has already been shown that higher levels of debt are associated with lower quality of life and burnout (8).

Our study found a high incidence of burnout across all 4 years of medical school which is consistent with previous work in medical students and residents (8, 17). Interestingly, feelings of burnout were not associated with either absolute or relative debt level, perhaps reflecting the fact that students are not in repayment. However, increased relative debt was associated with increased feelings of callousness towards others.

Despite the focus on student debt as a determinant of personal and professional outcomes, our study corroborates the well-documented phenomenon that gender, ethnicity, and marital status strongly influence personal and professional decision making (12). For instance, our study found that women had twice the odds of being concerned about managing and paying back educational debt, half the odds of being concerned that their debt had already influenced their specialty choice, nearly three times the odds of planning on working in an underserved area, and 1.6 times the odds of planning on working in an inner city area. Students identifying as Black or African American had half the odds of delaying having children because of medical student loan debt and four times the odds of planning to practice in an inner city area. Students identifying as Asian or Asian American had 1.6 times the odds of worrying that their educational debt had already impacted their specialty choice, 0.34 times the odds of being interested in practicing in a rural area, and on average selected a specialty with an annual salary $36,000 greater than their peers. Lastly, being married was protective against burnout and callousness and increased the odds of being interested in practicing in a rural area, but decreased the odds of planning to work in an inner city area. Students with children also had a significantly decreased odds of planning to work in inner city areas.

While our study paints a complex picture of how debt impacts medical students, there are significant limitations, including the low response proportion, use of a novel relative debt model, use of a questionnaire, and the nature of cross-sectional studies. First and foremost is the low overall response proportion. Thorough analysis of all of the included schools and students suggest that our study is representative of the national pool of medical schools, each included school individually, and therefore that our respondents are representative of the national medical student body. Notably, however, our sample does include more students attending private medical schools, slightly more students identifying as female, significantly more students identifying as white and thus lower numbers of minority students, and a nearly even distribution between students in the first 4 years of medical school (Tables 1–3). Our study also included students with significantly higher mean graduating debts than the national average for their year, with 4.30% of graduating respondents reporting no medical student debt, while nationally 14% graduated in 2012 without medical student debt. However, a pool of predominantly white, female debtors does not preclude the validity of our study, as our results are primarily analytic and not simply descriptive. Thus, a homogenous pool of respondents would weaken the power of our study, not provide positive bias for it.

Another potential limitation of our study is our creation of a relative debt model. The new relative debt model that we have proposed provides insight into why students with very different debt levels display the same behaviors associated with high debt. However, in creating the PECOAS we made several key assumptions. First, we assumed that the cost of attendance was nearly constant over the last few years, despite a compound annual growth rate in median 4-year cost of attendance of 5.8%. This created an artificial association between PECOAS and year in medical school by overestimating the savings of students who began prior to 2010 and underestimating the savings of those who began in 2010 or later, which had the potential to confound results. However, this association was accounted for in the multivariate analysis and actually trended any associations with the PECOAS towards the null. The estimated cost of attendance was also difficult to standardize as there is variation in how medical schools calculate cost of attendance. Our estimated cost of attendance includes tuition, fees, and health insurance, but does not include living expenses, which can vary widely based on geographical location and can be a substantial source of borrowing for many students. These problems with our PECOAS variable would tend to introduce random variation however, and thus all associations with the PECOAS concluded in these analyses were likely underestimations.

**Table 4:** Summary of respondent agreement, mean PECOAS values, and mean medical student loan debts

Question	Percent agree	Percent disagree	Mean PECOAS agree	Mean PECOAS disagree	*p*	Mean student loan debt agree	Mean student loan debt disagree	*p*
1) I have already or may in the future delay buying a house because of my educational debt	77.7	13.7	−0.31	0.17	<0.0001	124,000	76,000	<0.0001
2) I have already or may in the future delay getting married because of my educational debt	34.5	44	−0.3	−0.14	0.0001	125,000	106,000	0.0001
3) I have already or may in the future put off having children because of my educational debt	58.7	26.2	−0.34	−0.002	<0.0001	126,000	95,000	<0.0001
4) In the future I plan to practice in an underserved area	30.8	36.5	−0.26	−0.24	0.36	124,000	116,000	0.069
5) I am likely to practice in an inner-city area upon graduation from residency	32.8	31.4	−0.22	−0.29	0.041	121,000	116,000	0.16
6) Practicing in a rural area interests me	29.2	48.5	−0.31	−0.18	0.0014	115,000	116,000	0.72
7) Choose to become a physician again, if you were to revisit that choice	71.9	11.2	−0.21	−0.23	0.35	110,000	136,000	0.0001
8) My educational debt often causes me high levels of stress	50.8	27.2	−0.39	0.06	<0.0001	136,000	78,000	<0.0001
9) I worry about my ability to pay back my educational debt	68.6	20.7	−0.38	0.21	<0.0001	131,000	72,000	<0.0001
10) I am concerned about my ability to manage my educational debt	65.2	23.3	−0.37	0.15	<0.0001	135,000	70,000	<0.0001

Additionally, our study may be limited because of discordance between what our respondents report and what these respondents will actually choose in the future. For example, when the third- and fourth-year medical students report on intended specialty choice between February and May, there is a high likelihood that these students will ultimately match in their desired specialty. However, the responses of the first- and second-year students who lack clinical experience are far less likely to be accurate. Additionally, because of the competitive nature of some medical specialties, debt may be a secondary consideration to the ability of the student to match.

Lastly, it must be noted that the associations drawn from a cross-sectional study go both ways. Namely, that while absolute and relative debt may predict some of the outcomes of interest, so too may those outcomes predict debt levels. Our study attempted to account for this effect by explicitly including causal relations to debt in the study questions (e.g., ‘I have already or may in the future delay buying a house because of my educational debt’). Some of our statistical analyses do not include this safeguard, however. For example, our study found that students with more relative and absolute debt are more likely to choose a higher paying specialty or explicitly desire a higher income, respectively. The implication here is that debt influenced students towards their respective outcomes. However, the alternative explanation is that students who desire a higher paying specialty or higher explicit income are more likely to tolerate higher debt levels to do so. The truth is likely a combination of these two hypotheses, where some students feel the pressure of their debt, while others feel the security of their future incomes. Although the validity of alternative hypotheses to our conclusions may reduce the impact of interventions based on this study, it certainly does not invalidate them or reduce the urgency of the matter at hand.

In 2005, Jolly in Health Affairs questioned the sustainability of rising student debt when the median debt was $100,000 for public schools at $135,000 for private schools at interest rates of 2.82% (26). In 2006 the variable interest rate was replaced with a 6.8% fixed interest rate. In 2012 the interest rate subsidy for graduate and professional students was lost due to Congressional inaction, a decision that will cost the average medical student borrower $10,000–$20,000 over the life of their loans (1). Now in 2014 approximately one-third of students will graduate with more than $200,000 financed mostly through Federal Unsubsidized loans at an interest rate of 6.8% and Graduate Plus loans at an interest rate of 7.9%. Our study clearly suggests a multitude of associations between absolute and relative debt, gender, ethnicity, personal plans, and career choices. Although absolute recommendations are difficult to make, the data suggest that relative debt influences the decisions of future physicians and makes a case for all medical schools to reduce or control debt, particularly among those students who borrow the most. Efforts aimed at increasing diversity, reducing relative debt, and helping students to cope with or feel confident in their ability to manage debt may have a substantial influence on those students wavering between PC and HPNP specialties (13).

However, programs to help students cope with the current system may not go far enough. Reforms that create shorter paths to specific career options, such as those that rely on assessments of competency rather than time in training, are an excellent first step, but bolder innovations are needed (3, 4). These may include programs that financially benefit medical students for their valuable contributions, such as teaching assistant roles and patient education activities, which can begin to both lower the cost of education and reduce overall health care spending.
